# TM4SF1 Promotes Proliferation, Invasion, and Metastasis in Human Liver Cancer Cells

**DOI:** 10.3390/ijms17050661

**Published:** 2016-05-03

**Authors:** Yu-Kun Huang, Xue-Gong Fan, Fu Qiu

**Affiliations:** 1 Department of Infectious Diseases, Xiangya Hospital, Central South University, Changsha 410008, China; hyukun@hotmail.com (Y.-K.H.); dian1044@126.com (X.-G.F.); 2 Key Laboratory of Viral Hepatitis, Central South University, Changsha 410008, China; 3 Department of General Surgery, Xiangya Third Hospital, Central South University, Changsha 410013, China

**Keywords:** *TM4SF1*, liver cancer, proliferation, metastasis

## Abstract

Transmembrane 4 superfamily member 1 (*TM4SF1*) is a member of tetraspanin family, which mediates signal transduction events regulating cell development, activation, growth and motility. Our previous studies showed that *TM4SF1* is highly expressed in liver cancer. HepG2 cells were transfected with TM4SFl siRNA and *TM4SF1*-expressing plasmids and their biological functions were analyzed *in vitro* and *in vivo*. HepG2 cells overexpressing *TM4SF1* showed reduced apoptosis and increased cell migration *in vitro* and enhanced tumor growth and metastasis *in vivo*, whereas siRNA-mediated silencing of *TM4SF1* had the opposite effect. *TM4SF1* exerts its effect by regulating a few apoptosis- and migration-related genes including *caspase-3*, *caspase-9*, *MMP-2*, *MMP-9* and *VEGF*. These results indicate that *TM4SF1* is associated with liver tumor growth and progression, suggesting that *TM4SF1* may be a potential target for treatment of liver cancer in future.

## 1. Introduction

Hepatocellular carcinoma is a primary malignancy of the liver. It accounts for the third leading cause of cancer deaths worldwide, with over 600,000 people affected [1]. It mainly develops from chronic liver disease such as hepatitis B virus and hepatitis C virus infections. Emerging evidence has shown that there have been enormous changes of many signaling pathways during the development of liver cancer, with many surface proteins involved. These surface proteins have potential to become excellent therapeutic targets for liver cancer treatment.

The transmembrane-4 superfamily (TM4SF) is a group of cell-surface low molecular weight proteins that have four highly hydrophobic transmembrane domains. *TM4SF1* is an important member of the TM4SF. In particular, previous research showed that cervical cancer, lung cancer, squamous cell cancer, colon cancer, and breast cancer have elevated expression of *TM4SF1* mRNA, and that prostate cancer has elevated expression of *TM4SF1* protein [2]. Our previous studies showed that 86% of patients with hepatitis B virus-related hepatocellular carcinoma have overexpressed *TM4SF1* in their liver cancer cells, but that adjacent normal tissues and normal liver tissues had no measureable expression of *TM4SF1* [3]. Other studies reported that *TM4SF1* expression is closely related to the metastasis and recurrence of prostate cancer, non-small cell lung cancer, and breast cancer, and that *TM4SF1* expression is negatively associated with the survival of patients with squamous cell lung cancer [4]. In addition, some members of the TM4SF family (*TM4SF3*, *TM4SF5*, *cD151*, and *cD82*) have roles in the invasion and metastasis of liver cancer [5,6,7,8]. However, few studies have investigated the role of *TM4SF1* in liver cancer. Thus, the purpose of the present study was to examine the role of *TM4SF1* in regulating the proliferation, migration, and invasion of liver cancer cells.

## 2. Results

### 2.1. Effect of TM4SF1 on Apoptosis of HepG2 Cells

**Figure 1:**
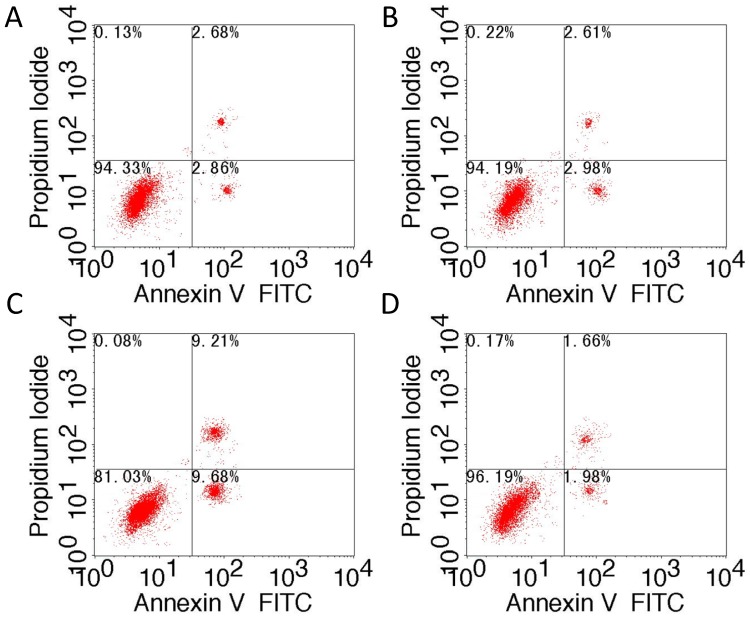

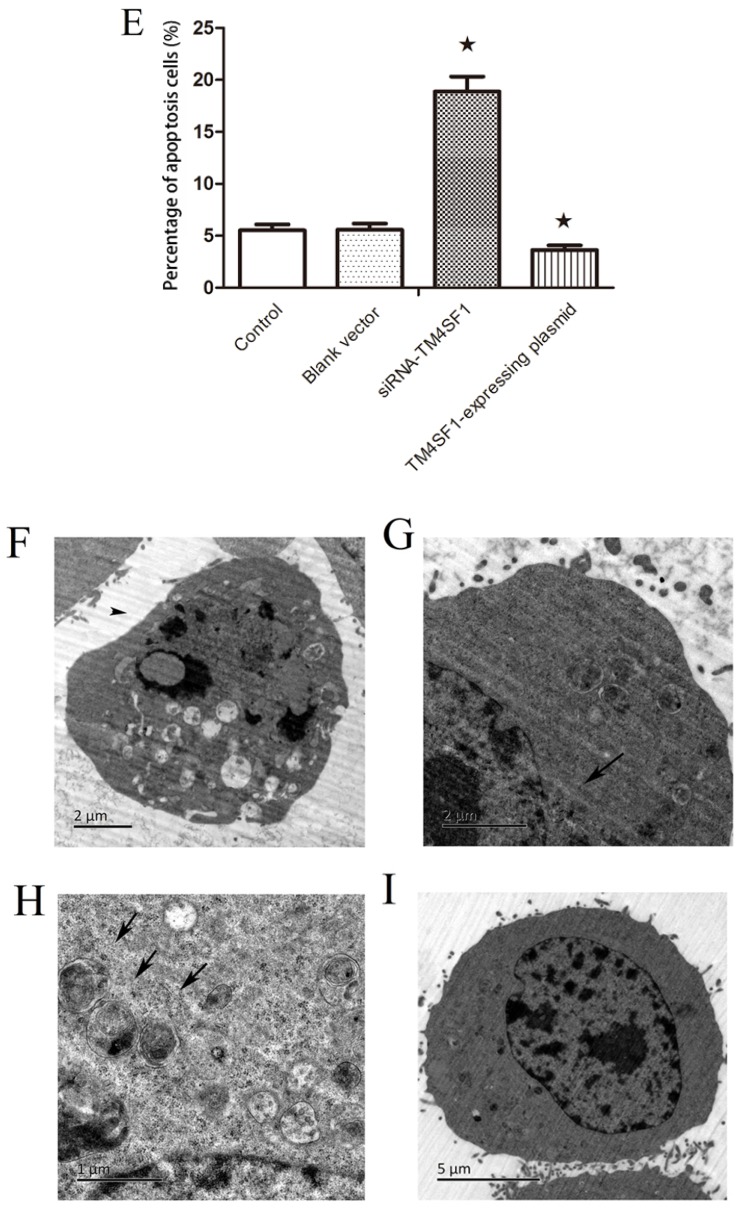
*TM4SF1* gene knockdown led to increased apoptosis and autophagy of HepG2 cells while *TM4SF1* overexpression reduced the apoptosis of cells. HepG2 cells were not transfected (**A**); transfected with blank vectors (**B**); transfected with siRNA-*TM4SF1* (**C**); or transfected with *TM4SF1*-expressing plasmids (**D**) and then harvested and processed for measurement of apoptosis by flow cytometry (**E**). Transmission electron microscopy was used to determine apoptosis and autophagy of HepG2 cells without transfection (**F**); transfected with blank vectors (**G**); transfected with siRNA-*TM4SF1* (**H**); or transfected with *TM4SF1*-expressing plasmids (**I**). Arrowhead, karyokinesis; Arrow, autophagosomes. Experiments were performed 3 times and similar findings were observed. ★ *p* < 0.01 *vs.* non-transfected HepG2 cells.

Cancer cells evolve various strategies to evade apoptosis by generating genetic mutations or epigenetic modifications in the key modulators of apoptosis pathways. Apoptosis may block metastatic dissemination by killing misplaced cells. Thus, apoptosis serves as an important process for inhibiting metastasis. To investigate effect of TM4SF1 on tumor cell apoptosis, TM4SF1 expression vector and siRNA were used to modulate expression of TM4SF1 in HepG2 cells (Figures S1 and S2). HepG2 cells were not transfected (Figure 1A), transfected with blank vectors (Figure 1B), transfected with siRNA-TM4SF1 (Figure 1C), or transfected with TM4SF1-expressing plasmids (Figure 1D) and then harvested and processed for measurement of apoptosis by flow cytometry (Figure 1E). TM4SF1 gene knockdown led to increased apoptosis of cells relative to controls (*p* < 0.01) while TM4SF1 overexpression reduced the apoptosis of cells relative to controls (*p* < 0.01). Transmission electron microscopy was used to determine apoptosis and autophagy of HepG2 cells without transfection (Figure 1F), transfected with blank vectors (Figure 1G), transfected with siRNA-TM4SF1 (Figure 1H), or transfected with TM4SF1-expressing plasmids (Figure 1I). Transmission electron microscopy studies have shown that only a small number of control cells exhibited karyokinesis and had autophagosomes. TM4SF1 overexpressing cells had uniform cytoplasms, evident nucleoli, and no apoptotic cells or autophagosomes. Cells transfected with siRNA-TM4SF1 had obvious pyknosis, and large numbers of apoptotic bodies and autophagosomes.

### 2.2. TM4SF1 Affects HepG2 Cells Migration

**Figure 2:**
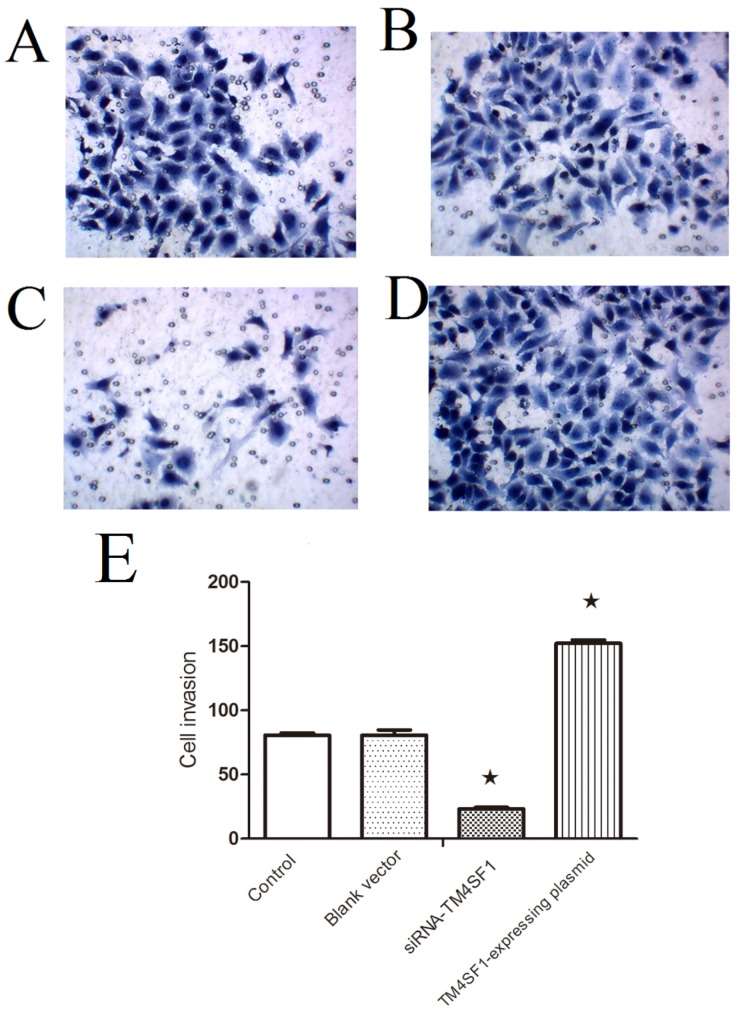
*TM4SF1* gene knockdown led to reduce the migration of HepG2 cells and *TM4SF1* overexpression increased migration of cells. Cells without transfection (**A**); transfected with blank vectors (**B**); transfected with siRNA-*TM4SF1* (**C**); or transfected with *TM4SF1*-expressing plasmids (**D**) were harvested and seeded into Transwell chambers (5 × 10^5^ cells/chamber) for evaluation of migration (**E**) under an inverted microscope (200×) for identification of hematoxylin-positive cells. The experiment was performed four times. ★ *p* < 0.01 *vs*. non-transfected HepG2 cells.

To assess the role of *TM4SF1* on HepG2 cells migration, *TM4SF1* expression vector and siRNA were used to modulate expression of *TM4SF1* in HepG2 cells and then measured migration of HepG2 cells. Cells without transfection (Figure 2A), transfected with blank vectors (Figure 2B), transfected with siRNA-*TM4SF1* (Figure 2C), or transfected with *TM4SF1*-expressing plasmids (Figure 2D) were harvested and seeded into Transwell chambers for evaluation of cell migration. As shown in Figure 2E, *TM4SF1* gene knockdown led to reducing the migration of cells relative to controls (*p* < 0.01) and *TM4SF1* overexpression increased migration of cells relative to controls (*p* < 0.01).

### 2.3. Effect of TM4SF1 on Expression of Cancer-Related Proteins in HepG2 Cells

**Figure 3:**
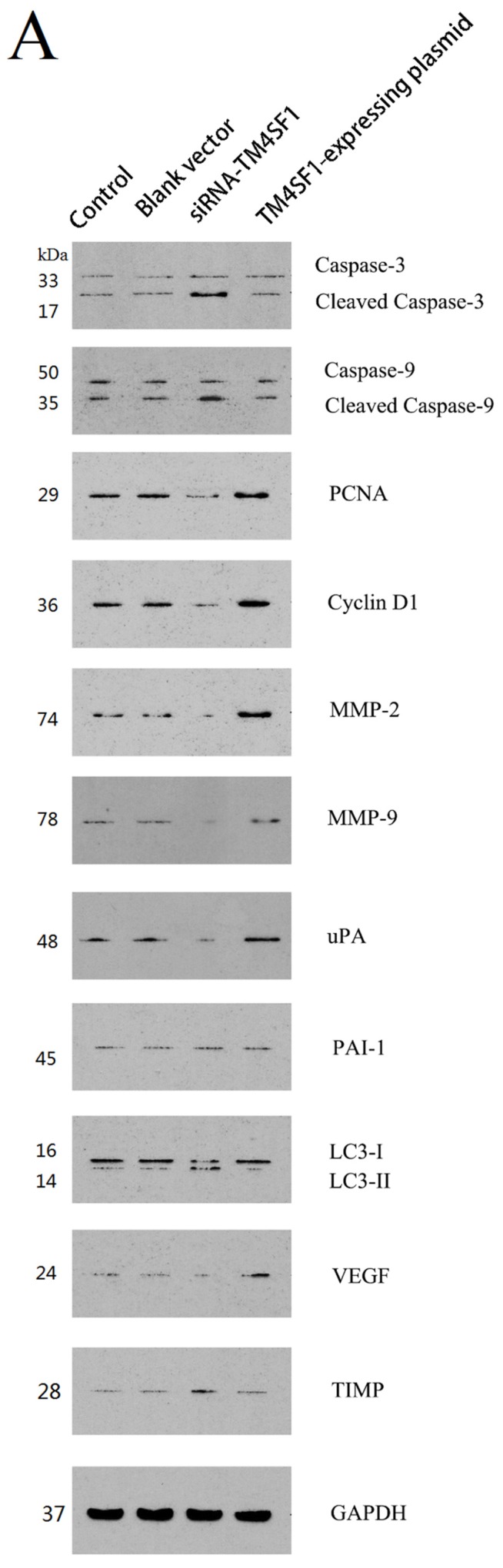

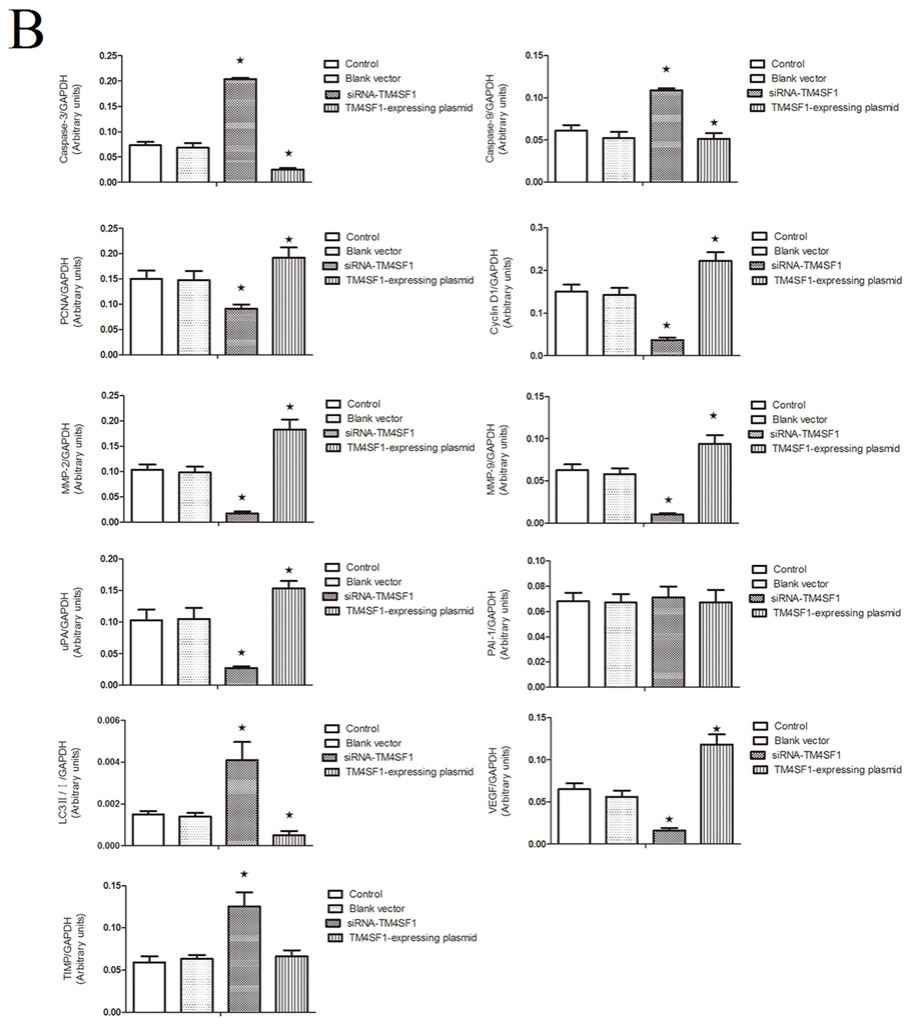
*TM4SF1* overexpression reduced the protein expression of *caspase-3*, *caspase-9*, and LC3-II in HepG2 cells, but increased expression of *PCNA*, cyclin D1, *MMP-2*, *MMP-9*, *uPA*, and *VEGF*. *TM4SF1* gene knockdown increased the protein expression of *caspase-3*, *caspase-9*, and LC3-II, but decreased expression of *PCNA*, cyclin D1, *MMP-2*, *MMP-9*, *uPA*, and *VEGF*. (**A**) Protein levels of *caspase-3*, *caspase-9*, *PCNA*, cyclin D1, *MMP-2*, *MMP-9*, *uPA*, *PAI-1*, LC3, *VEGF*, *TIMP* and GAPDH were determined by immunoblot analyses of whole-cell lysates with the respective Abs; (**B**) Densitometric quantification of protein levels were normalized to GAPDH levels. The experiment was performed three times. ★ *p* < 0.01 *vs*. non-transfected HepG2 cells.

To illustrate the role of *TM4SF1* in cancer-related proteins, *TM4SF1* expression vector and siRNA were used to modulate expression of *TM4SF1* and then measured cancer-related proteins in HepG2 cells. As shown in Figure 3, *TM4SF1* overexpression reduced the protein expression of *caspase-3*, *caspase-9*, and LC3-II relative to controls, but led to increased expression of *PCNA*, cyclin D1, *MMP-2*, *MMP-9*, *uPA*, and *VEGF* relative to controls (*p* < 0.01 for all comparisons). *TM4SF1* gene knockdown increased the protein expression of *caspase-3*, *caspase-9*, and LC3-II relative to controls, but led to decreased expression of *PCNA*, cyclin D1, *MMP-2*, *MMP-9*, *uPA*, and *VEGF* relative to controls (*p* < 0.01 for all comparisons).

### 2.4. TM4SF1 Regulates Tumor Growth in Vivo by Modulating Cell Apoptosis

**Figure 4:**
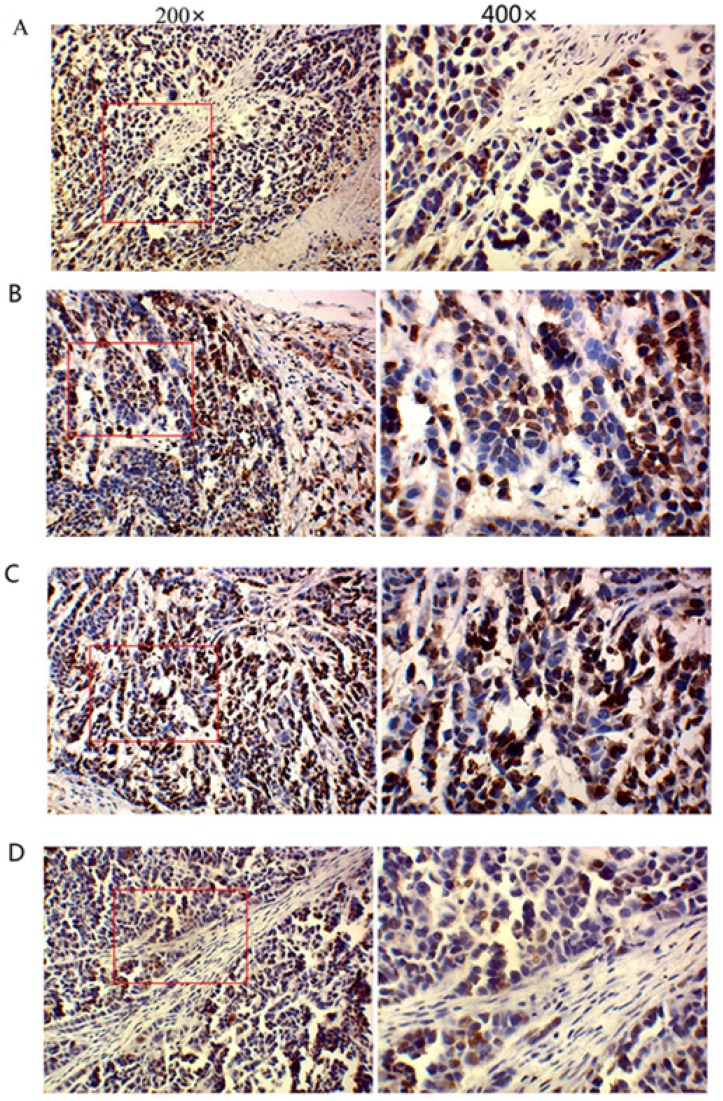

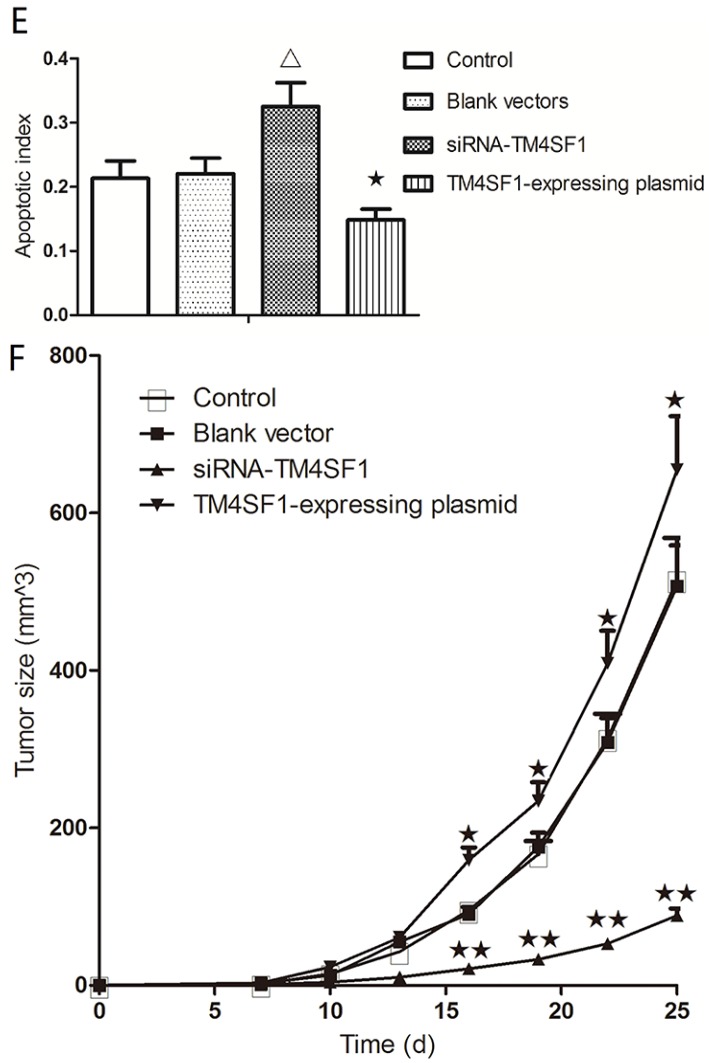
*TM4SF1* regulates tumor growth *in vivo* by modulating cell apoptosis. HepG2 cells that were not transfected (**A**); or transfected with blank vectors (**B**); siRNA-*TM4SF1* (**C**); or *TM4SF1*-expressing plasmids (**D**) were harvested and inoculated into nude mice; and the apoptotic index (**E**) and tumor size were measured (**F**). Tumor volume was calculated as: (maximum diameter) × (minimum diameter)^2^ × 0.52. Δ *p* < 0.01 *vs.* non-transfected HepG2 cells; ★ *p* < 0.01 *vs.* non-transfected HepG2 cells; ★★ *p* < 0.001 *vs.* non-transfected HepG2 cells.

To determine the molecular mechanism of how TM4SF1 regulates tumor growth, we focused on the cell apoptosis; it is well known that decreased susceptibility to apoptosis plays an important role in tumor growth [9]. Transfection with siRNA-TM4SF1 significantly reduced the number of cells relative to controls and transfection with TM4SF1-expressing plasmids increased the number of cells relative to controls (Figure S3). Nude mice were given subcutaneous injection of HepG2 cells without transfection (Figure 4A), or transfected with blank vectors (Figure 4B), siRNA-TM4SF1 (Figure 4C), or TM4SF1-expressing plasmids (Figure 4D). As shown in Figure 4E, HepG2 cells with TM4SF1 overexpression showed less cell apoptosis (based on TUNEL staining) than injection with control cells at 25 days (*p* < 0.01). Injection with HepG2 cells transfected with siRNA-TM4SF1 led to greater cell apoptosis than injection with control cells at 25 days (*p* < 0.01). Subcutaneous injection of nude mice with HepG2 cells that were transfected with TM4SF1-expressing plasmids led to significantly larger tumors than injection with control cells on day 16, 19, 22, and 25 (*p* < 0.01 for all comparisons). Injection with HepG2 cells transfected with siRNA-TM4SF1 led to significantly smaller tumors than injection with control cells at day 16, 19, 22, and 25 (*p* < 0.001 for all comparisons) (Figure 4F).

### 2.5. TM4SF1 Has a Significant Effect on Regulation of Several Cancer-Related Proteins in Vivo

**Figure 5:**
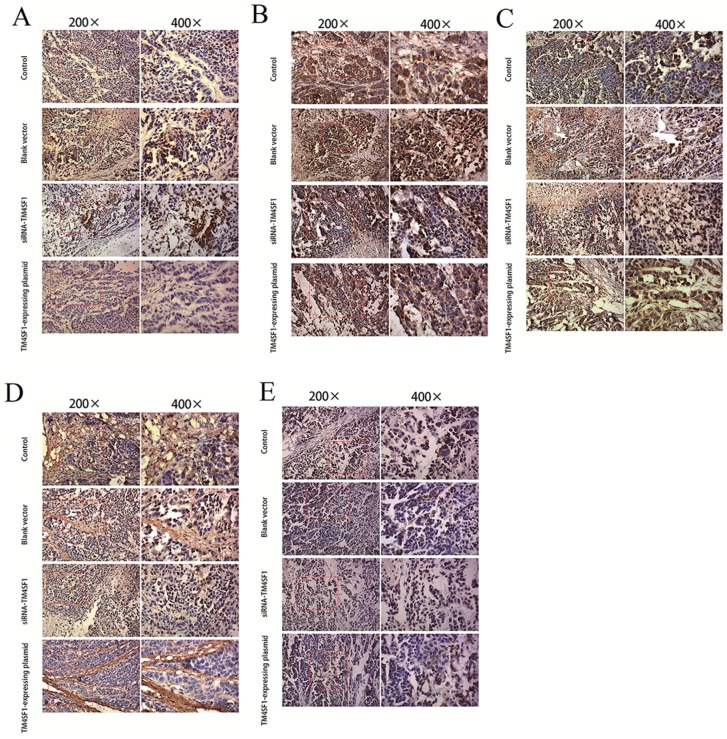

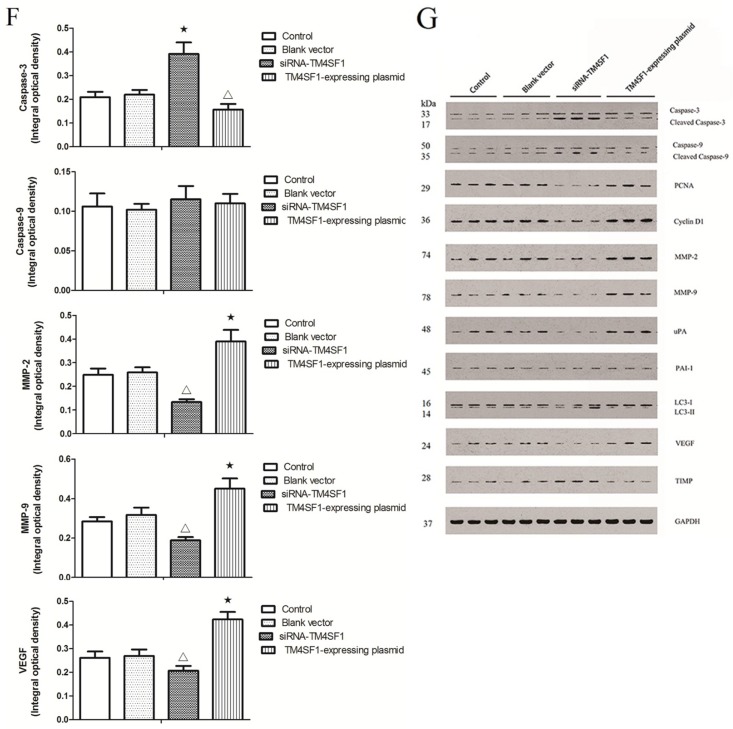

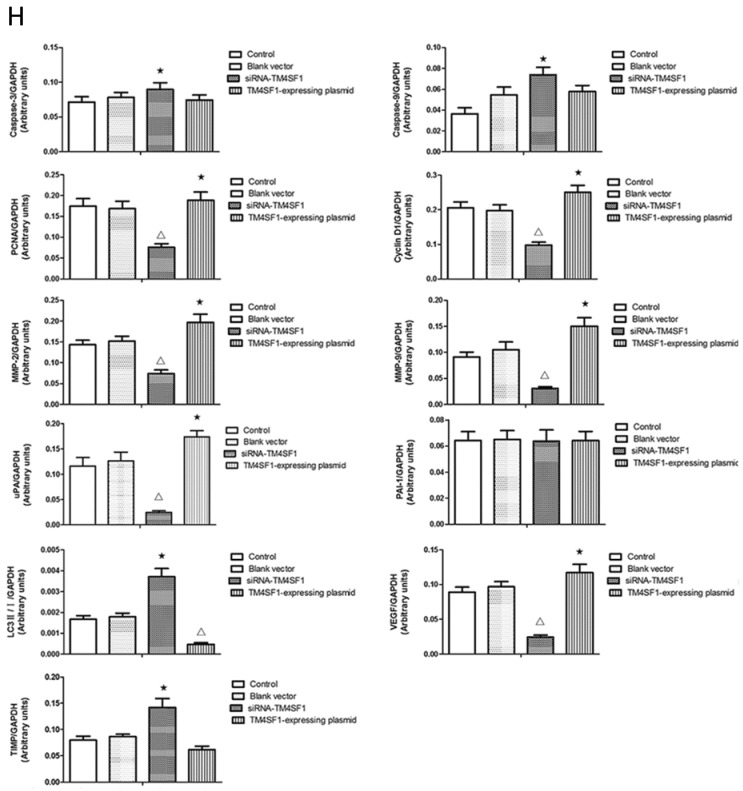
*TM4SF1* has a significant effect on regulation of several cancer-related proteins *in vivo*. Nude mice were injected with HepG2 cells that were transfected with siRNA-*TM4SF1*, *TM4SF1*-expressing plasmids, blank vectors, or cells without transfection, and immunohistochemistry was performed 25 days later to measure expressions of *caspase-3* (**A**); *caspase-9* (**B**); *MMP-2* (**C**); *MMP-9* (**D**); and *VEGF* (**E**). (**F**) The integrated optical density of *caspase-3*, *caspase-9*, *MMP-2*, *MMP-9*, and *VEGF*-positive cells; (**G**) Western blot analyses of these proteins were also performed on harvested tissues; (**H**) Densitometric quantification of protein levels were normalized to GAPDH levels. The experiment was performed three times. Δ *p* < 0.01 *vs.* non-transfected HepG2 cells; ★ *p* < 0.01 *vs*. non-transfected HepG2 cells.

To determine the molecular mechanism of how *TM4SF1* promotes liver tumor growth and progression, we focused on several cancer-related proteins, which are known to play a major role in the development and progression of liver cancer [10]. Nude mice were injected with HepG2 cells that were transfected with siRNA-*TM4SF1*, *TM4SF1*-expressing plasmids, blank vectors, or cells without transfection, and immunohistochemistry was performed 25 days later to measure expressions of *caspase-3* (Figure 5A), *caspase-9* (Figure 5B), *MMP-2* (Figure 5C), *MMP-9* (Figure 5D), and *VEGF* (Figure 5E). As shown in Figure 5F, at 25 days after subcutaneous injection of nude mice with HepG2 cells that were transfected with *TM4SF1*-expressing plasmids, tumor expression of *MMP-2*, *MMP-9*, and *VEGF* were significantly higher, but tumor expression of *caspase-3* and *caspase-9* were significantly lower, relative to injection with control cells (*p* < 0.01 for all comparisons) based on immunohistochemical analysis and/or Western blot analysis. At 25 days after injection of HepG2 cells that were transfected with siRNA-*TM4SF1*, tumor expression of *MMP-2*, *MMP-9*, and *VEGF* were significantly lower, but tumor expression of *caspase-3*, *caspase-9*, and *TIMP* were higher, relative to injection with control cells (*p* < 0.01 for all comparisons) (Figure 5G,H).

## 3. Discussion

Cancer cells are characterized by increased proliferation and reduced apoptosis. Cyclin D1 promotes passage through phase G1 of the cell cycle and is overexpressed in liver cancer; overexpression of cyclin D1 appears to promote cancer cell invasion [11]. In liver cancer patients, high proliferating cell nuclear antigen (*PCNA*) expression is associated with increased involvement of blood vessels, and reduced postoperative disease-free survival time [12]. The results of present study showed that cells with upregulated *TM4SF1* had increased expression of cyclin D1 and *PCNA* and that silencing of *TM4SF1* reduced the expression of these two genes and also inhibited the proliferation and growth of HepG2 cells. This suggests that in the pathogenesis of liver cancer, *TM4SF1* upregulates cyclin D1 and *PCNA* and thereby promotes the growth, proliferation, and invasion of cancer cells.

Our results showed that upregulation of *TM4SF1* significantly inhibited the apoptosis of HepG2 cells, and that silencing of *TM4SF1* expression with siRNA induced the apoptosis of these cells. We also found that upregulation of *TM4SF1* inhibited the expression of *caspase-3* and *caspase-9* in HepG2 cells and growth of transplanted tumors and that silencing of *TM4SF1* increased the expression of *caspase-3* and *caspase-9* in HepG2 cells and growth of transplanted tumors. Thus, we speculate that *TM4SF1* upregulation inhibits apoptosis and induces abnormal proliferation of liver cancer cells by downregulating *caspase-3* and *caspase-9*, and that this leads to the onset and progression of liver cancer.

Recent studies indicate that autophagy may inhibit the onset and progression of numerous cancers. For example, cells with stable transfection of *beclin-1* had increased autophagy and reduced tumorigenesis [13]. Downregulation of beclin-1 reduces cell autophagy and prolongs the life cancer cells, leading to increased development of cancers [14,15]. Our results showed that transfection of HepG2 cells with *TM4SF1*-expressing plasmids significantly increased *TM4SF1* expression, and that this markedly inhibited the autophagy of HepG2 cells, as indicated by the presence of fewer autophagosomes and reduced LC3II expression. Silencing of *TM4SF1* increased the autophagy of HepG2 cells, and this was accompanied by significant increases in the number of autophagosomes and expression of LC3II. Thus, inhibition of the autophagy of cancer cells may be one of the mechanisms underlying *TM4SF1*-induced tumorigenesis.

Although autophagy can inhibit tumorigenesis, other studies showed that autophagy can also promote the survival of cancer cells. For example, when tumor growth overwhelms angiogenesis, there may be focal ischemia and hypoxia; under these stressful conditions, which often occur at the center of cancers where new blood vessels do not form, the survival of cancer cells depends on catabolism during autophagy [16]. The present study of nude mice was a preliminary examination of the role of autophagy in the early phase of tumorigenesis (25 days after subcutaneous injection of HepG2 cells). The results showed that upregulation of *TM4SF1* inhibited the autophagy of liver cancer cells and increased the susceptibility to tumorigenesis, and that downregulation of *TM4SF1* markedly promoted the autophagy of cancer cells and decreased the susceptibility to tumorigenesis. Thus, we speculate that in the early phase of liver cancer, downregulation of *TM4SF1* plays an important role in the promotion of tumorigenesis.

Some findings suggest that there is crosstalk between autophagy and apoptosis, and that *caspase-3* and *caspase-9* may mediate this effect. *Caspase-9* may form complexes with ATG7 and induce the formation of LC3-II and thereby promote autophagy [17]. *Caspase-3* may be a molecular switch that mediates the crosstalk between autophagy and apoptosis, and activated *caspase-3* can promote secretion of autophagic vacuoles [18]. Our results showed that upregulation of *TM4SF1* downregulates the expression of *caspase-3* and *caspase-9*, and inhibits the apoptosis and autophagy of HepG2 cells, thereby promoting cell proliferation and facilitating tumorigenesis. On the contrary, downregulation of *TM4SF1* upregulates the expression of *caspase-3* and *caspase-9*, and activates apoptosis and autophagy of HepG2 cells, thereby suppressing cell proliferation and tumorigenesis. Thus, we speculate that in the early pathogenesis of liver cancer, *TM4SF1* has a central role in the inhibition of apoptosis and autophagy that is mediated through its effects on *caspase-3* and *caspase-9*.

Vascular endothelial growth factor (*VEGF*) is a highly specific mitogen of the vascular endothelium that increases the permeability of microvessels, blocks the degeneration of newly generated blood vessels, increases glucose transportation by the vascular endothelium, promotes the division and proliferation of the vascular endothelium, and facilitates the migration of endothelial cells [19,20,21]. There is evidence that *VEGF* promotes the secretion of some enzymes that facilitate the metastasis of cancers, and that overexpression of *VEGF* can induce *MMP-2* and *MMP-9*, which may be a major mechanism underlying the invasion and metastasis of highly invasive cancers [22,23].

*MMP-9* disrupts the basement barrier and promotes the migration of capillary endothelial cells to initiate cancer angiogenesis [24]. The *MMP-2* (located at 16q21) is a major component of the MMP family and has extensive distribution. This protein degrades the ECM and thereby promotes the migration of cancer cells across the ECM and basement barrier and the subsequent metastasis of cancer cells through connective tissues [25,26,27,28]. The substrates of *MMP-2* and *MMP-9* are mainly the skeletal components of the basement membrane, such as type IV and type V collagen. *TIMP* can irreversibly bind to MMP, inhibit MMP activity, block degradation of the ECM, and thereby inhibit the invasion and metastasis of cancers [29]. The results of our studies of HepG2 cells and transplanted tumors showed that *TM4SF1* promoted the expression of *uPA*, *MMP-2*, and *MMP-9*, and that silencing of *TM4SF1* inhibited the expression of *uPA*, *MMP-2*, and *MMP-9* and elevated *TIMP* expression. Our results also indicated that *TM4SF1* had no effect on the expression of *PAI-1*. We speculate that *TM4SF1*-mediated upregulation of *uPA*, *MMP-2*, and *MMP-9* increases the degradation of ECM by cancer cells, leading to invasion and metastasis of cancer cells. Silencing of *TM4SF1* appears to restore the balance between MMP and *TIMP*, increase the inhibition of MMP by *TIMP*, reduce degradation of the ECM, thus inhibiting the invasion and metastasis of cancer cells.

*TM4SF1* may promote the invasion and metastasis of cancers through one or more mechanisms. First, it may promote the vascular endothelial cells in the cancer to initiate angiogenesis, which indirectly promotes cancer cell invasion and metastasis. On the other hand, it may strengthen the interaction between cancer cells and the ECM, which facilitates the invasion and metastasis of cancer cells [30]. In addition, *TM4SF1* overexpression is also involved in the formation of pseudopodia in cancer cells, and this facilitates the invasion and metastasis of cancer cells [31,32], and *TM4SF1* has recently been reported to stimulate breast cancer cell invasion and migration through PI3K/AKT/mTOR pathway [33]. It should be noted that *TM4SF1* is highly expressed in liver cancer, and liver cancer patients with *TM4SF1* overexpression have worse five-year survival rates than those with low *TM4SF1* expression [34], supporting our results.

*TM4SF1* expression is also elevated in lung cancer, pancreatic cancer, liver cancer, and cervical cancer, leading to its classification as a tumor-associated antigen [35,36,37,38]. After injection of a human-mouse chimeric monoclonal antibody against *TM4SF1*, 22.2% (4/18) of patients with breast cancer, colon cancer, or non-small cell lung cancer produced antibodies against antibody, and these aggregated around cancer cells [39]. Our results confirmed that *TM4SF1* was closely related to the migration and invasion of HepG2 cells. Overexpression of *TM4SF1* in HepG2 cells significantly increased the migration of cells across the Matrigel membrane and promoted the growth of transplanted tumors. Silencing of TM4SFl markedly reduced the migration of HepG2 cells across the Matrigel membrane and inhibited the growth of transplanted tumors. This suggests that silencing of TM4SFl expression should be considered as a potential new strategy for the therapy of liver cancer.

## 4. Experimental Section

### 4.1. Materials

Human liver cancer cells (HepG2 cells) were provided by the Department of Infectious Diseases of the Affiliated Xiangya Hospital of Central South University. *TM4SF1*-expressing plasmids were prepared by Shanghai Genepharma Co., Ltd. (Shanghai, China). Lipofectamine^®^ 2000 and the Trizol reagent were from Invitrogen (Carlsbad, CA, USA); RPMI-1640, trypsin, fetal bovine serum (FBS), and G418 were from Gibco (Grand Island, NY, USA); monoclonal antibodies against *TM4SF1*, *MMP-2*, *PAI-1*, *uPA*, *TIMP*, and *PCNA* were from Abcam (Cambridge, UK); monoclonal antibodies against *caspase-9* and *caspase-3* were from Bioss (Woburn, MA, USA); monoclonal antibodies against LC3I/II and cyclin D1 were from Cell Signaling Technology (Danvers, MA,USA); monoclonal antibodies against *MMP-9* were from ProteinTech Group (Chicago, IL, USA); monoclonal antibodies against GAPDH were from Santa Cruz Biotechnology (Dallas, TX, USA); Transwell assays and matrigel were from BD Biosciences (San Jose, CA, USA); ultraSensitive TM-SP was from Fuzhou Maxim Biotech Co., Ltd. (Fuzhou, China); and ECL+ was from Amersham (Piscataway, NJ, USA).

### 4.2. Plasmid Construction

The open reading frames (ORFs) of h*TM4SF1* were cloned from HEK293 cell cDNA using the primer pairs h*TM4SF1*-F and h*TM4SF1*-R, and h*TM4SF1* based on the h*TM4SF1* (GenBank accession no. 4071 and Refseq: NM_014220) sequences. The primer pairs for *TM4SF1* were 5′-ATGTGCTATGGGAAGTGTGCAC-3′ (forward), and 5′-TGGTTGTCGTTATACTGACGATT-3′ (reverse). pGBKT7-h*TM4SF1* was constructed by cloning h*TM4SF1* into the expression vector pGBKT7 (Clontech, California, CA, USA), which encoded the full-length *TM4SF1* fused to the GAL4 DNA-binding domain for yeast two-hybrid screening. pcDNA3.1-Myc-h*TM4SF1* was obtained by the respective cloning of h*TM4SF1* genes into the mammalian expression vector pcDNA3.1-Myc (Invitrogen, California, CA, USA) to express h*TM4SF1* fused to a N-terminal Myc epitope tag. pEGFP-N1 empty vector (Clontech) encoding enhanced green fluorescent protein (EGFP), and was used as a control. pGEM-h*TM4SF1* was constructed by cloning h*TM4SF1* into the transcription vector pGEM-4Z (Promega, Madison, AL, USA).

### 4.3. Cell Culture and Plasmid Transfection

HepG2 cells were maintained in RPMI1640 medium that contained 100 mL/L FBS, 100 U/mL penicillin, and 100 μg/mL streptomycin at 37 °C in a humidified environment with 5% CO_2_. Opti-MEM® I was used to dilute blank vectors, *TM4SF1*-expressing plasmids, and Lipofectamine^®^ 2000, followed by incubation at room temperature for 5 min. The diluted blank vectors and *TM4SF1*-expressing plasmid solutions were independently mixed with Lipofectamine^®^ 2000, followed by incubation at room temperature for 20 min. The resultant solution was transferred into plates containing HepG2 cells, followed by incubation for 72 h. Cells were harvested for real-time PCR, flow cytometry, transmission electron microscopy, Transwell migration assay, MTT assay, Western blotting, and subcutaneous injection into *Foxn1*^−/−^ nude mice.

### 4.4. Gene Silencing with siRNA

Three pairs of siRNAs that targeted *TM4SF1* were designed and synthesized: (i) siRNA-*TM4SF1*-497: 5′- GCGAUGCUUUCUUCUGUAUTT-3′ (forward), 5′-AUACAGAAGAAAGCAUCGCTT-3′ (reverse); (ii) siRNA-*TM4SF1*-733: 5′-GGCUCUUGGUGGAAUUGAATT-3′ (forward), 5′-UUCAAUUCCACCAAGAGCCTT-3′ (reverse); and (iii) siRNA-*TM4SF1*-813: 5′-GCUCUCACCAACAGCAAUATT-3′ (forward), 5′- UAUUGCUGUUGGUGAGAGCTT-3′ (reverse). Scrambled siRNA (forward: 5′-UUCUCCGAACGUGUCACGUTT-3′; reverse: 5′-ACGUGACACGUUCGGAGAATT-3′) was synthesized as a control. Opti-MEM^®^ I was used to dilute these siRNAs or Lipofectamine^®^ 2000, followed by incubation at room temperature for 5 min. The resultant siRNA was mixed with Lipofectamine® 2000, incubated at room temperature for 20 min, and then transferred onto plates containing HepG2 cells. Cells were maintained for 24 h and then harvested for real-time PCR, flow cytometry, transmission electron microscopy, Transwell migration assay, MTT assay, Western blotting, and subcutaneous injection into *Foxn1*^−/−^ nude mice (see below).

### 4.5. Real-Time PCR

Total RNA was extracted from HepG2 cells transfected with siRNA-*TM4SF1*, *TM4SF1*-expressing plasmids, and blank vectors and from HepG2 cells without transfection by use of the Trizol reagent according to manufacturer’s instructions. The MyIQ real-time PCR system (Bio-Rad, Hercules, CA, USA) was used to measure mRNA expression of *β-actin* (housekeeping gene) and *TM4SF1*. The primer for *β-actin* was 5′-CATTAAGGAGAAGCTGTGCT-3′ (forward), 5′-GTTGAAGGTAGTTTCGTGGA-3′ (reverse) and the primer for *TM4SF1* was 5′-AAGGGGGAGAAAACCTAGCA-3′ (forward), 5′-CCAGCCCAATGAAGACAAAT-3′ (reverse).

### 4.6. Flow Cytometry

HepG2 cells that were transfected with siRNA-TM4SF1, TM4SF1-expressing plasmids, and blank vectors and HepG2 cells without transfection were subjected to Annexin V-FITC/PI staining according to the manufacturer’s instructions. After washing in PBS, cells were re-suspended in binding buffer and cell density was adjusted to 5 × 105/mL. Then, 195 μL of cell suspension was mixed with 5 μL of Annexin V-FITC, followed by incubation at room temperature for 10 min. After one wash in PBS, cells were re-suspended in 190 μL of binding buffer, followed by addition of 10 μL of 20 µg/mL propidium iodide. Finally, cells were washed and then analyzed using a flow cytometer. Percentage of apototosis cells (%) = (number of Annexin V^+^PI^−^ and Annexin V^+^PI^+^ cells)/total cells × 100%.

### 4.7. Transmission Electron Microscopic Examination

The morphology of HepG2 cells that were transfected with siRNA-*TM4SF1*, *TM4SF1*-expressing plasmids, and blank vectors and HepG2 cells without transfection were examined using a transmission electron microscope at the Xiangya School of Medicine Electron Microscope Facility, Central South University, China. The cells were fixed in phosphate-buffered 2.5% glutaraldehyde for 24 h, postfixed in phosphate-buffered 2% osmium tetroxide for 2 h, dehydrated in ascending concentrations of acetone, infiltrated over 24 h with Spurr’s resin, and observed using a Hitachi-7700 transmission electron microscope (Ibaraki, Japan).

### 4.8. Transwell Migration Assay

In the Transwell migration assay, HepG2 cells transfected with siRNA-*TM4SF1*, *TM4SF1*-expressing plasmid, or blank vectors and HepG2 cells without transfection were seeded into the upper Transwell chambers (5 × 10^4^ cells) and maintained in serum free medium. In the lower chamber, medium containing 150 mL/L FBS was added, followed by incubation at 37 °C with 5% CO_2_ for 24 h. The upper chambers were taken out and the inner cells were removed from the upper chambers, which was then washed twice and fixed in 95% ethanol, followed by hematoxylin staining. Cells were observed under an inverted microscope. Five fields were randomly selected and positive cells were classified as invasive.

### 4.9. Animal Study

Foxn1^−/−^ nude mice (6 to 8 weeks, Department of Animal Experiments, Central South University) were used in all animal studies. National Institutes of Health Guidelines for Care and Use of Laboratory Animals were observed. HepG2 cells transfected with siRNA-TM4SF1, TM4SF1 expressing plasmid, or blank vector and HepG2 cells without transfection were subcutaneously inoculated into Foxn1^−/−^ nude mice (1 × 106 HepG2 cells/mouse). The tumor volume was measured as maximum longest diameter × minimum shortest diameter2 × 0.52. At 25 days after subcutaneous injection, mice were sacrificed and the transplanted tumors were collected for analysis. All studies were approved by the Institutional Review Board of Third Xiangya Hospital, Central South University, China (4 March 2015, No: 2015-S035).

### 4.10. Western Blotting

HepG2 cells transfected with siRNA-*TM4SF1*, *TM4SF1*-expressing plasmids, blank vectors and cells without transfection, and the transplanted tumors of nude mice were harvested. Total protein was extracted from cells and tissues for measurement of protein expression. Cell extracts were prepared using a lysis buffer containing 20 mM HEPES (pH 7.4), 0.5% Triton X-100, 150 mM NaCl, 12.5 mM β-glycerophosphate, 50 mM NaF, 1 mM DTT, 1 mM sodium orthovanadate, 2 mM EDTA, 1 mM PMSF, and protease inhibitor cocktail (Roche Applied Science, Indianapolis, IN, USA). Protein concentration of cell extracts was determined by the Bradford protein reagent (Bio-Rad), using BSA as a standard. Equal amounts of cell extracts were resuspended in Laemmli loading buffer (Bio-Rad), boiled and subjected to SDS-polyacrylamide gel electrophoresis to separate proteins on 4%–20% polyacrylamide minigels (Invitrogen). Proteins were electrotransferred to Immobilon-P membranes (Millipore, Billerica, MA, USA), membranes were blocked with Tris-buffered solution (TBS) with 0.1% Tween 20 (TBS-T), containing 5% non-fat dry milk (Bio-Rad), and probed for 20 h at 4 °C with the corresponding primary antibodies, such as the monoclonal antibody against *TM4SF1*, *caspase-3*, *caspase-9*, *PCNA*, cyclin D1, *MMP-2*, *MMP-9*, *uPA*, *PAI-1*, LC3, *VEGF*, *TIMP* and GAPDH. After washing five times in TBS-T, membranes were incubated with the corresponding anti-rabbit, anti-mouse, or anti-goat secondary IgG-HRP conjugates diluted at 1:12,000 in TBS-T/5% milk, before being washed three times; bands were revealed by incubation with enhanced chemiluminescence reagents (ECL+) followed by exposure to X-ray films. Densitometric analysis of intensities of the protein bands from three independent experiments was performed using the Quantity One software (Bio-Rad). Levels of *TM4SF1*, *caspase-3*, *caspase-9*, *PCNA*, cyclin D1, *MMP-2*, *MMP-9*, *uPA*, *PAI-1*, LC3, *VEGF*, and *TIMP* were normalized to those of GAPDH, and data were expressed as arbitrary units.

### 4.11. Terminal dUTP Nick End-Labeling (TUNEL) Staining

HepG2 cells transfected with siRNA-TM4SF1, TM4SF1-expressing plasmids, blank vectors and cells without transfection, and transplanted tumors of nude mice were harvested and processed for the measurement of apoptosis. The terminal dUTP nick end-labeling (TUNEL) assay was performed using the TdT-FragEL TM DNA Fragmentation Detection kit (Calbiochem/Oncogene Research Products, Cambridge, MA, USA) according to the manufacturer’s instructions. Briefly, 4 μm sections from the paraffin-embedded samples were dewaxed with xylene and hydrated using graded alcohols, and the specimens were treated with 20 mg/mL proteinase K for 5 min and with 0.6% H2O2 in methanol to eliminate endogenous peroxidase activity. Afterward, the sections were treated with the TDT enzyme and immersed in a biotinylated nucleotides solution. Apoptotic cells were detected using streptavidin–peroxidase conjugate followed by diaminobenzidine staining. All sections were observed under the same magnification, light source, brightness, color saturation, gain, and contrast. Five fields were randomly selected from each section, and images were processed with Motic Fluo 1.0 image analysis software (Motic China Group Co., Ltd., Guangzhou, China). TUNEL-positive cells had yellow-brown granules in the nucleus. After adjusting optical density, the apoptotic index was calculated by division of the number of labeled cells by the total number of cells in six high power fields (original magnification: 400×).

### 4.12. Immunohistochemistry

Immunohistochemistry was performed according to manufacturer’s instructions by use of the SP method (UltraSensitive TM-SP). In brief, sections were routinely deparaffinized and treated with methanol that contained 3% hydrogen peroxide for 30 min. Then, sections were blocked in normal serum (50 μL/section) for 10 min at room temperature, and incubated with the primary antibody (*caspase-3*, *caspase-9*, *MMP-2*, *MMP-9* and *VEGF*) or PBS (negative control) at 4 °C overnight. After incubation with biotin-conjugated secondary antibody (50 μL/section) for 10 min at room temperature, 50 μL of streptavidin-peroxidase was added, followed by incubation at room temperature for 10 min. Sections were then treated with freshly prepared DAB solution for 3−10 min depending on the staining intensity (observed by microscopy). Sections were washed three times in PBS (3 min each) between steps. After staining, sections were counterstained with hematoxylin for 2 min and then treated with ethanol in HCl. Sections were washed in water, dehydrated in ethanol, transparentized in xylene, mounted with neutral gum, and observed under a light microscope. Positive cells had yellow-brown granules in the cytoplasm. Endothelial cells with yellow-brown granules in the cytoplasm were *VEGF*-positive cells; non-endothelial cells with yellow-brown granules in the cytoplasm were *caspase-9*- or *MMP-2*-positive cells, and monocytes with yellow-brown granules were positive for *caspase-3*. The IOD values for *caspase-3*, *caspase-9*, *MMP-2*, *MMP-9*, and *VEGF* cells were determined independently.

### 4.13. Statistical Analysis

Statistical analysis was performed using the GraphPad Prism 5 program for Windows (Graphpad Software, San Diego, CA, USA). Statistical differences between experimental groups were evaluated by a one-way ANOVA with repeated measures, followed by *post hoc* comparisons with Tukey’s multiple paired comparison test. Values are expressed as mean ± SD. A *p* ≤ 0.05 was considered significant.

## 5. Conclusions

Taken together, our results showed that overexpression of *TM4SF1* significantly increased the proliferation and tumorigenesis of liver cancer cells. Moreover, upregulation of *TM4SF1* downregulates the expression of pro-apoptotic genes (*caspase-3* and *caspase-9*), upregulates the expression of genes related to cell proliferation and cell cycle progression (cyclin D1 and *PCNA*), inhibits cell apoptosis and autophagy, and increases cell proliferation. In addition, when cancer cells with *TM4SF1* overexpression were injected into nude mice, this increased the expression of genes related to angiogenesis (*uPA*, *MMP-2*, *MMP-9* and *VEGF*), decreased the expression of *TIMP* (an inhibitor of MMP), and led to promotion of angiogenesis and tumor growth. Based on these findings, *TM4SF1* appears to enhance the invasion of cancer cells by several mechanisms (Figure S4). Silencing of *TM4SF1* expression downregulates the expression of genes related to regulation of the cell cycle and cell proliferation (cyclin D1 and *PCNA*), upregulates the expression of genes related to apoptosis and autophagy (*caspase-3* and *caspase-9*). Silencing of *TM4SF1* also increases the expression of *TIMP* (an inhibitor of MMP), inhibits the expression of pro-angiogenic genes (*uPA*, *MMP-2*, *MMP-9*, and *VEGF*) and suppresses the proliferation, invasion and metastasis of cancer cells. Thus, inhibition of *TM4SF1* expression may be a useful strategy to inhibit tumor growth and to reduce the migration and invasion of cancer cells.

## Supplementary Materials

Supplementary materials can be found at http://www.mdpi.com/1422-0067/17/5/661/s1.

## Abbreviations

*TM4SF1*	transmembrane 4 superfamily member 1
*MMP*	matrix metalloproteinases
*TIMP*	tissue inhibitor of matrix metalloproteinases type
*PCNA*	proliferating cell nuclear antigen
*uPA*	urokinase-type plasminogen activator
*PAI-1*	plasminogen activator inhibitor-1
LC3	microtubule-associated protein light chain 3
*VEGF*	vascular endothelial growth factor
ATG	autophagy-related protein
ASP	apoptosis-specific protein
