# Supplementary material for: TM4SF1 Promotes Proliferation, Invasion, and Metastasis in Human Liver Cancer Cells
Source: Int J Mol Sci. 2016 May 3;17(5):661. doi: 10.3390/ijms17050661 (PMC4881487; doi:10.3390/ijms17050661)
Supplement: Supplementary file 1 [file ijms-17-00661-s001.pdf]

Supplementary Materials: TM4SF1 Promotes Proliferation, Invasion, and Metastasis in Human Liver Cancer Cells

Yu-Kun Huang, Xue-Gong Fan and Fu Qiu

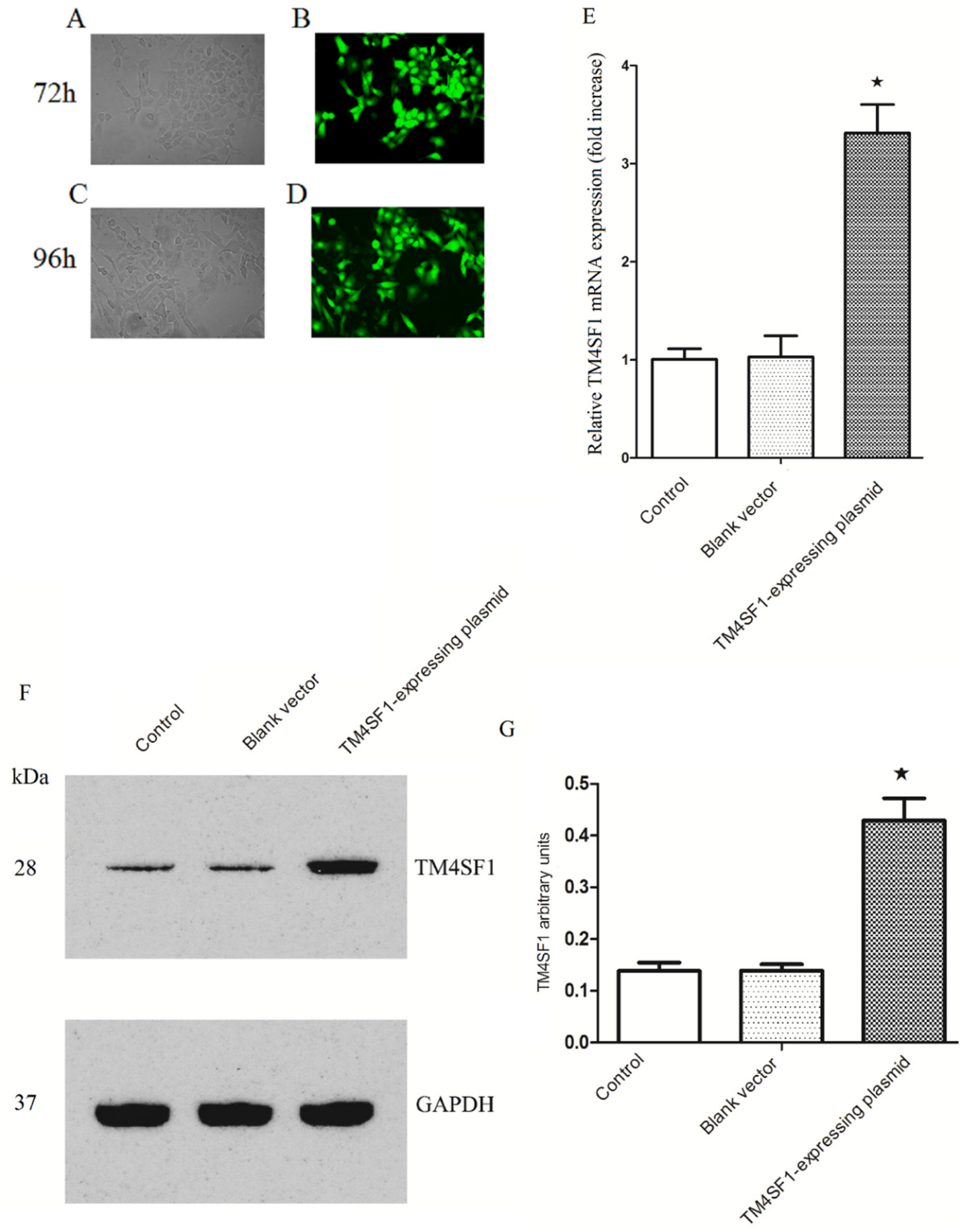

Figure S1. Expression of TM4SF1 in HepG2 cells after transfection with TM4SF1-expressing plasmids. HepG2 cells were transfected with TM4SF1-expressing plasmids, maintained in 5% CO_2 at 37 °C for 72 h and 96 h, and then observed by fluorescence microscopy (400×) and light microscopy (400×). At 72 h (**A**,**B**) and 96 h (**C**,**D**), the total cell number (**A**,**C**) and number of fluorescent cells (**B**,**D**) were determined. The results indicate that transfection

efficiency (fluorescent cells/total cells) was greater than 80% at both time points. HepG2 cells were transfected with TM4SF1-expressing plasmids or blank vectors, and real-time PCR and Westernblotting were used to measure expression of mRNA (**E**) and protein (**F**); Non-transfected HepG2 cells were controls. Densitometric quantification of levels of TM4SF1 (**G**) was performed for three independent experiments, and protein expression of the indicated proteins normalized to levels of GAPDH was presented as arbitrary units (mean ± SD). Transfection with TM4SF1-expressing plasmids led to significantly higher expression than transfection with blank vectors or controls (★ $p < 0.01$).

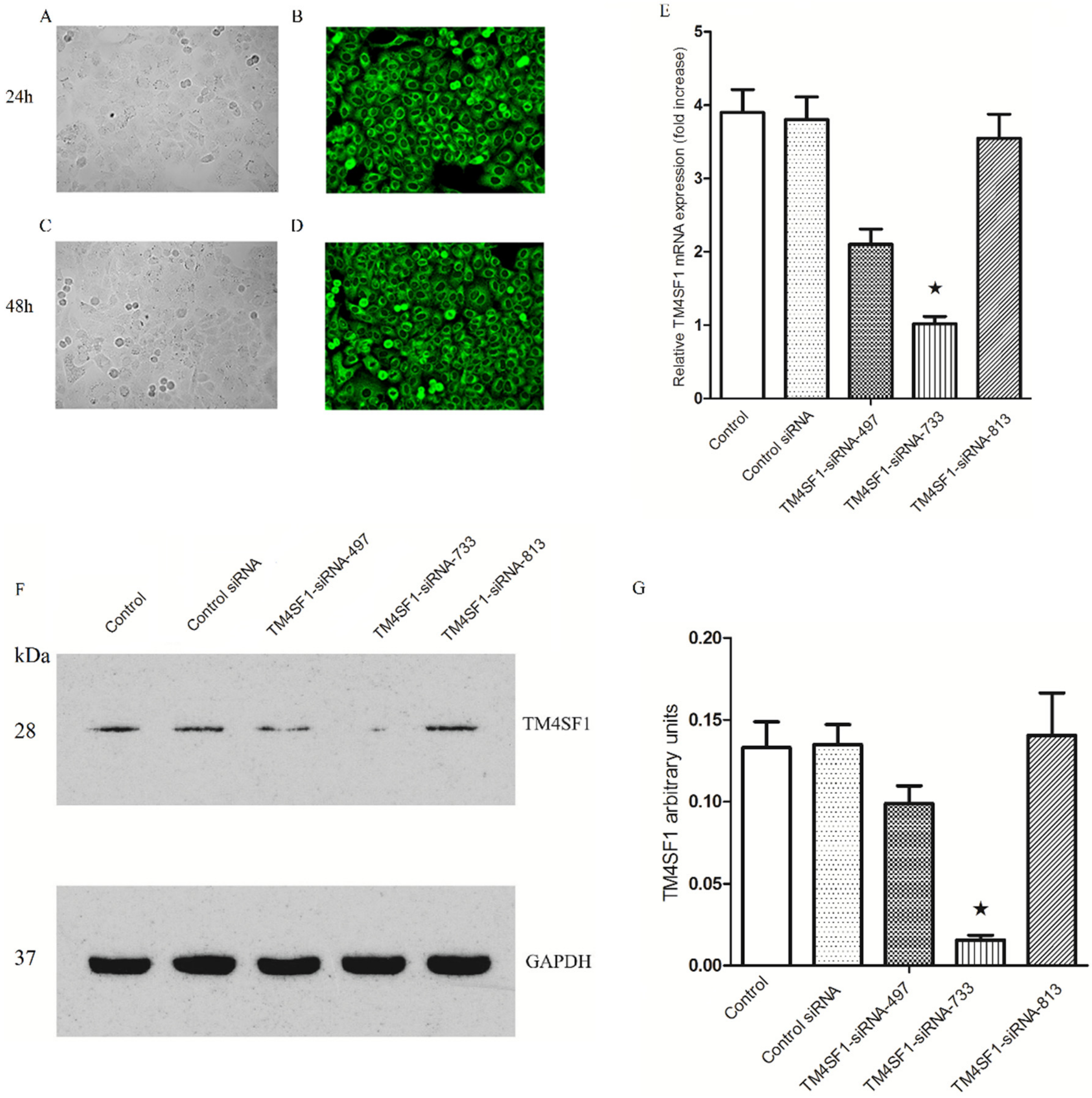

Figure S2. Expression of TM4SF1 in HepG2 cells after transfection with TM4SF1-siRNAs. HepG2 cells were transfected with TM4SF1-siRNA, maintained in 5% CO_2 at 37°C for 24 h and 48 h, and observed by fluorescence microscopy (400×) and light microscopy (400×). At 24 h (**A**,**B**) and 48 h (**C**,**D**), the total cell number (**A**,**C**) and the number of fluorescent cells (**B**,**D**) were determined. The results indicate that transfection efficiency (fluorescent cells/total cells) was greater than 80% at both time points. HepG2 cells were transfected with different TM4SF1-siRNAs (TM4SF1-siRNA-497, -733, and -813) or control siRNA. Non-transfected HepG2 cells were controls. Real-time PCR and Western blotting were used to measure expression of mRNA (**E**) and protein (**F**); Densitometric quantification of levels of

TM4SF1 (**G**) was performed for three independent experiments, and protein expression of the indicated proteins normalized to levels of GAPDH was presented as arbitrary units (mean ± SD). Protein and mRNA expression were lower after transfection with TM4SF1-siRNA-733 (a TM4SF1-siRNA) relative to non-transfected cells and cells transfected with TM4SF1-siRNA-497, TM4SF1-siRNA-813, and control siRNA (★ $p < 0.001$).

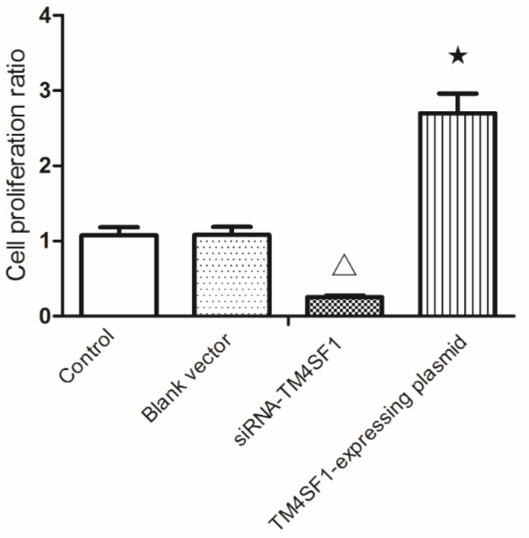

Figure S3. Effect of TM4SF1 expression on proliferation of HepG2 cells. Cells transfected with siRNA-TM4SF1, TM4SF1-expressing plasmids, or blank vectors, and those without transfection were harvested and cultured for 5 days, followed by MTT assays to evaluate cell proliferation. The number of HepG2 cells after transfection with siRNA-TM4SF1 (Δ) was significantly reduced compared with other groups ($p < 0.01$). The number of HepG2 cells after transfection with TM4SF1-expressing plasmids (★) was dramatically increased compared with other groups ($p < 0.01$). The experiment was performed 4 times and similar findings were observed.

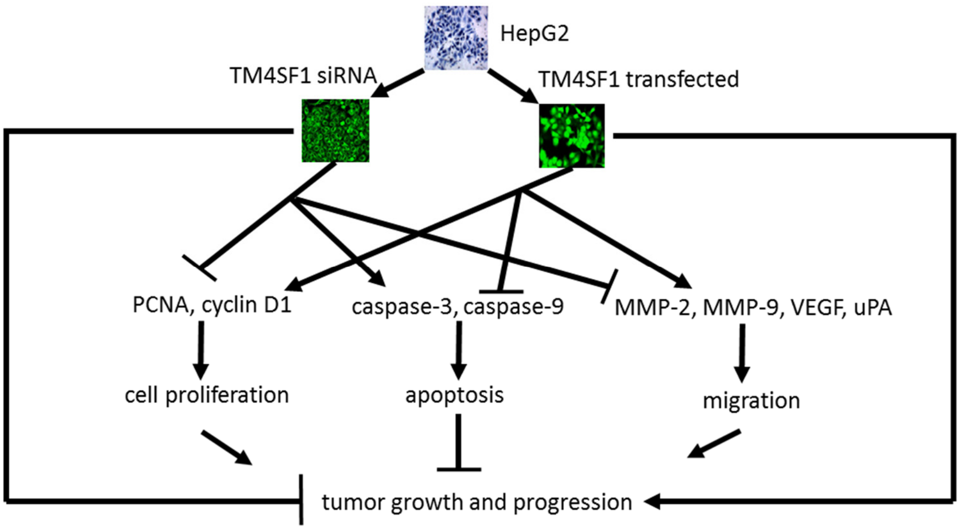

Figure S4. TM4SF1 plays an important role in tumor growth and progression of HCC. HepG2 cells over-expressing TM4SF1 showed reduced the expression of caspase-3 and caspase-9, but led to increased expression of PCNA, cyclin D1, MMP-2, MMP-9, VEGF, and uPA, whereas siRNA-mediated silencing of TM4SF1 had the opposite effect.
